# Patient-reported outcome measures as an outcome variable in sports medicine research

**DOI:** 10.3389/fspor.2022.1006905

**Published:** 2022-11-02

**Authors:** Alison R. Snyder Valier, Kellie C. Huxel Bliven, Kenneth C. Lam, Tamara C. Valovich McLeod

**Affiliations:** ^1^Department of Athletic Training, Arizona School of Health Sciences, A.T. Still University, Mesa, AZ, United States; ^2^School of Osteopathic Medicine in Arizona, A.T. Still University, Mesa, AZ, United States; ^3^Department of Interdisciplinary Health Sciences, Arizona School of Health Sciences, A.T. Still University, Mesa, AZ, United States

**Keywords:** patient-centered care, clinical outcomes assessment, athletic training, methodology, rehabilitation research

## Abstract

Injury prevention and rehabilitation research often address variables that would be considered clinician-oriented outcomes, such as strength, range of motion, laxity, and return-to-sport. While clinician-oriented variables are helpful in describing the physiological recovery from injury, they neglect the patient perspective and aspects of patient-centered care. Variables that capture patient perspective are essential when considering the impact of injury and recovery on the lives of patients. The inclusion of patient-reported outcome measures (PROMs) as dependent variables in sports medicine research, including injury prevention and rehabilitation research, provides a unique perspective regarding the patient's perception of their health status, the effectiveness of treatments, and other information that the patient deems important to their care. Over the last 20 years, there has been a significant increase in the use of PROMs in sports medicine research. The growing body of work gives opportunity to reflect on what has been done and to provide some ideas of how to strengthen the evidence moving forward. This mini-review will discuss ideas for the inclusion of PROMs in sports medicine research, with a focus on critical factors, gaps, and future directions in this area of research. Important elements of research with PROMs, including instrument selection, administration, and interpretation, will be discussed and areas for improvement, consideration, and standardization will be provided.

## Introduction

Recently, there have been efforts to give patients a voice in both clinical care and research. A focus on patient-centered care has permeated to sports medicine, providing opportunities for patients and families to be active collaborators in the development and implementation of their comprehensive care plan (1). In regards to sports medicine research, efforts to include patient-reported outcome measures (PROMs), a source of patient-oriented evidence, have been ongoing and are highlighted by calls by professional organizations and government entities, such as the Patient-Centered Outcomes Research Institute (PCORI) (2). A proliferation of published research on PROMs in the past decade further supports the increased utilization of these measures among sports medicine researchers. A database search using the terms *patient reported outcome measures* and *sports medicine* identified 562 citations in the years 2000–2009. This number increased to 4,250 between 2010 and 2019, with 3,217 publications already between 2020 and 2022. Unfortunately, issues still exist in the use of PROMs in sports medicine research, such as inconsistency and variability in choosing and implementing PROMs within investigations (3). This can introduce bias and influence the research findings of individual studies, as well as subsequent secondary analysis when conducting systematic reviews or meta-analyses if authors are aiming to synthesize a body of evidence, in which PROMs are not used similarly across individual studies (4).

Historically, sports medicine research, including research specific to injury prevention and rehabilitation, has focused on clinician-oriented outcomes, including strength, range of motion, and functional tests. While some patient-oriented outcomes have accompanied clinician-oriented outcomes in sports medicine research, the focus has been on variables such as patient perception of pain. Although an important outcome, pain offers a limited perspective on the patient's function and overall health status and is often assessed with a numeric pain rating scale that is unidimensional in nature and does not provide detailed information regarding the patient's perception of pain. Neglecting other important dimensions of a patient's health, including quality of life (e.g., Pediatric Quality of Life Inventory), psychological readiness (e.g., Anterior Cruciate Ligament-Return to Sport after Injury scale), and injury-related fear (e.g., Tampa Scale of Kinesiophopia) across different domains of disablement (e.g., activity, participation, and environmental factors) limits the comprehensiveness of a patient-centered approach. Current recommendations to embrace the biopsychosocial model of health requires the assessment of multidimensional health constructs including physical, psychological, interpersonal and contextual factors in a dynamic model (5). The inclusion of a broader set of multidimensional health constructs may allow clinicians and researchers to better understand the patient experience, appreciate patient perception of health, and assess additional domains of contemporary disablement models (6). While there has been a move to include more PROMs in research, there are still certain methodological issues with the inclusion of PROMs into sports injury prevention and rehabilitation research.

Current evidence suggests that many studies do not adequately use PROMs within sports medicine research (Table 1) (7–9). The adequate use of PROMs has been defined as using a PROM that measures what it claims to (content validity), has undergone appropriate statistical validation (reliability, validity, responsiveness), and is used to evaluate patients with conditions similar to those used in its validation (7). In a review of 349 articles, almost half were identified as having at least one irregularity or potential problem in PROM selection (9). For example, a significant number of studies used a PROM that was developed for a different patient population or used two or more PROMs containing identical questions, which suggests issues with instrument selection. Similarly, recent analyses of randomized controlled trials (RCT) in sports medicine (7, 8) also noted inadequate use of PROMs in more than half of studies. Instrument selection was a concern, with PROMs being used in populations they were not validated in, as was instrument administration, with studies not reporting the research protocol. Further, another challenge for sports medicine researchers is PROM interpretation given that there is limited information to guide interpretation across the large number of instruments available and there is an evolution of the science of meaningful change (11–14). Evidence suggests the appropriate use of PROMs for a research question of interest is associated with larger treatment effects as compared to studies with inadequate PROM use (7), highlighting the need to educate researchers on proper PROM selection and use to reduce variability and increase consistency between studies and better capture treatment effects within studies. Therefore, the purpose of this mini-review is to aid sports medicine researchers in the selection, administration, and interpretation of PROMs. In addition, national and international efforts to improve the inclusion of PROMs into rehabilitation research will be discussed.

**Table 1:** Irregularities noted in studies of PROMs in sports medicine research (7–10).

**Potential problems**	**Possible solutions**
Using PROM developed for a different patient population	Choose the PROM that most closely resembles the conditions and patients being studied
Used for conditions other than the intended condition	Consider a generic PROM or choose one that fits the condition of interest
Using two or more PROMs with similar questions	Choose only the PROM that best fits the patient and condition to reduce redundancy, conflict across items, and patient and administrator burden
Aggregating domain scores to create a composite	Report the results as described in the PROM development and validation study. Use only domain scores if indicated
Exclusion of domains or items	Analyze all domains and items
Lack of content validity for specific patient populations	Assess content validity for that population. Choose a different PROM
Adaption of scale scores when results are reported	Present the data as described in the development or validation study. Do not rescale scores to percentages
Recall bias	Limit questions asking about function or health status prior to the initiation of treatment or interventions. Fully describe how data were collected within the manuscript and whether in real time or by recall

## Instrument selection

Sports medicine researchers integrating PROMs may find it challenging to decide which outcomes are most appropriate to use given the large number of PROMs available (10, 15–18). Patient-reported outcome measures can be designed as generic in focus which makes them applicable to evaluate health broadly in healthy or injured populations, or they can be designed to be specific to particular types of populations, regions, or conditions. Length is another consideration with some PROMs including a single item, like the Single Assessment Numeric Evaluation (SANE) (19), and others including multiple-items, such as the Lower Extremity Functional Scale (LEFS) (20). Use of generic vs. specific and single-items vs. multi-item PROMs will influence who the instruments can be administered to, time burden on researcher and patient, and depth and relevance of the information obtained from a population (21). Unfortunately, PROM selection is not a simple task and can be approached in different ways. A common approach is to vet a number of different PROMs according to general guidelines and identify the best fit for the study. Recent efforts to establish guidelines and checklists for including PROMs in research, particularly clinical trials, are helpful and should be used early in study development (22–25). For example, general guidelines to follow when selecting a PROM should: (1) identify relevant PROM domains of interest that align with research questions, (2) consider disease or condition-specific, population-specific, and region-specific PROMs that are likely to be influenced by the therapy or intervention being studied, (3) evaluate the psychometric properties (reliability, validity and responsiveness) of PROMs in the population being studied, and (4) consider practicalities of using the PROM in the study, such as respondent burden and mode of administration (17, 21, 24, 26, 27).

When identifying relevant PROM domains, frameworks, such as disablement models, may be helpful in choosing outcomes of interest most critical to patients (6). While pain is a common impairment studied for its importance to patients, researchers should consider other body structure and function impairments (e.g., fatigue, strength) and additional domains, such as function in terms of activity limitations and participation restrictions (e.g., throwing, running, playing sports, attending school) and psychological readiness. Selecting PROMs most likely to be influenced by the study's intervention is encouraged, and these tend to be disease or condition-specific, population-specific, and region-specific PROMs. Multidimensional PROMs that evaluate multiple social constructs, such as HRQOL, are important to understand in the context of the study's intervention, but are likely influenced by more aspects of the patient's life than the research study (24, 25). Therefore, using PROMs that evaluate HRQOL alone as an outcome is not advised if new interventions are being studied (25). Next, researchers should consider the PROM's psychometric properties. Specifically, researchers should consider how and in whom the PROM was developed (28), assess if the included items are relevant to the intended population, and determine if validity, reliability, and responsiveness of the PROM has been established for the intended population (10, 15–18, 29). Too often, modifications to PROMs are made to fit the population or study (e.g., wording altered, items removed or added, scoring adjusted), which compromises the scale's validity. In circumstances when researchers modify a PROM for their study, it is important to specify the modification in the protocol and recognize the psychometric properties may be compromised (25, 27). Validation of the modified PROM is suggested. Consideration of all of these factors may help to ensure that the selected PROMs are appropriate to for the population and to address the study aims and more likely to capture changes in health over time.

Another approach to selecting a PROM that researchers may find beneficial is to seek PROM recommendations from organizations vested in sports medicine research. Typically, these organizations have used experts to vet outcomes, ensure adequate measurement properties, and encourage synergy amongst researchers in the field. For example, the American Academy of Orthopedic Surgeons (AAOS) established a registry program in 2017 in collaboration with multiple specialty societies (30). One valuable result of a registry is that it can facilitate research aimed at a specific cause, such as increased patient safety, improving patient outcomes, and promoting best practices (30). The AAOS currently has five registries, including joint replacement, fracture and trauma, musculoskeletal tumor, shoulder and elbow, and spine, that offer resources and opportunities to access or contribute to research efforts (30).

Registries, often include required and suggested data elements and administration time points (30), also referred to as common data elements (CDE). A CDE is a set of variables, including PROMs, that are recommended in research relevant to the specific condition. The idea of the CDE is that it promotes consistency across studies in the way data are collected. Greater consistency can lead to more efficient cross-study comparisons and primary analyses as well as greater ability to perform secondary analyses, such as meta-analyses or systematic reviews. Another example of a collaborative, standardized data collection effort is the National Institute of Neurological Disorders and Stroke (NINDS) who has published several CDEs (31), including two related to traumatic brain injury and sport related concussion (32, 33). Selecting PROMs based on data sets that include CDEs not only streamlines the decision-making process but also creates opportunity to collaborate and align with research at a larger scale and for greater impact. However, the establishment of registries and CDEs is relatively recent and one may not exist yet for the condition or population of interest for all those engaged in sports medicine research.

Finally, given the importance of PROM selection, the COnsensus-based Standards for the selection of health Measurement INstruments (COSMIN) group has created a toolkit for sports medicine researchers interested in conducting patient-oriented research (4). The COSMIN toolkit is designed to support the selection of PROMs and provides numerous resources including a taxonomy to better define measurement properties of PROMs, a checklist to evaluate the quality of studies on measurement properties of PROMs, and a databases of studies about the measurement properties of PROMs.

## Instrument administration

After an appropriate PROM is selected, it is equally important to develop a protocol for how and when PROM administration will occur to ensure alignment with the research questions while also being cognizant of the administrative burden (2, 3, 25, 34). Topics to consider when developing the PROM administration protocol for a sports medicine study include establishing standardized time points for PROM administration, training the individuals who administer the PROM, and mode of PROM administration (3, 9, 25, 34).

### Establishing standardized time points for PROM administration

Decisions about when to administer a PROM is a common challenge for researchers because there are no established best practice or standardized time point(s) to guide research design. Common time points for PROM administration, which often represent the minimum required to answer research questions, are at the beginning (initial evaluation) and end (discharge) of an injury rehabilitation or prevention intervention. Comparing outcomes between these two time points enables researchers to examine patients' perceptions about the effectiveness of interventions and establish the time course of treatment for a specific condition or population. However, to ensure the research is relevant to the real-world, careful consideration should be given to aligning administration to time points that are clinically relevant to the patient population and intervention, which may benefit from the insight of patient populations (3, 35, 36). The PROM selected for use may have instructions or wording of questions that can be used to inform administration time points, such as when questions ask patients to answer based on their experiences and perceptions over a time period (e.g., the past week, the previous month), or in comparison to a prior state (e.g., pre-injury level). These reflection periods must be considered when determining administration time points.

Further, while a simple strategy is to administer at the start and end of an intervention, there is evidence to suggest that administrating PROMS at intermittent time points has a clinical benefit to patient outcomes. For example, Werneke et al. (37) used a therapeutic outcomes database to examine the time points of PROM administration that resulted in the highest functional status at discharge from rehabilitation in patients treated for non-specific lumbar impairments. Of the 140,336 patients with completed PROMs, 83,101 (59%) did so only at the beginning and end of therapy and had slightly lower functional outcomes than patients who completed interim PROMs. The administration of an interim PROM, regardless of the time point, led to higher functional status outcomes at discharge compared to no interim administration. Similarly, two or more interim PROMs resulted in better outcomes than one interim PROM. The best result in patients' functional status outcomes occurred when at least one interim PROM was administered during the first 2 weeks after initial evaluation (37). This study highlights the importance of interim time points in promoting positive patient outcomes. What is unknown, yet a relevant consideration for future outcomes researchers, is what led to the better outcomes when intermittent PROMs were used. In theory, use of intermittent PROMs provides an opportunity to interject patient voice into the care process and for clinicians to respond and possibly adjust the strategy based on patient input. However, researchers and patients are often blinded to clinical findings during data collection to reduce bias and to add a higher level of control. More discussion is needed related to navigating research methods and designs used to collect patient-reported data in clinical research and whether point-of-care approaches (38) with less blinding may be appropriate for some research questions and study designs, especially when inclusion of the patient is a key element to the research. Further, identifying a standardized set of specific administration time points that can be shared across sports medicine researchers may strengthen the body of evidence from this research community.

### Individuals administering PROMs

A component of sports medicine research protocols often overlooked involves the individuals who administer the PROMs to patients. These individuals may not have an extensive research training background or may not have been involved with the development of the research design, and they have reported a lack of clarity on alignment of the PROM to the research questions and expectations for how to administer these measures (25). Individuals responsible for administering PROMs require training related to the study protocol, purpose of the PROMs, informed consent procedures, and anticipated participant questions to provide firm guidance on executing the study protocol to support participant compliance and the situation of missing or incomplete data (25). This area of the study protocol development should involve the individuals administering the PROMs as research personnel and include practical aspects of administration such as workflow adjustments, identifying the primary administrator, and designing a secondary plan to maintain continuity in PROM collection if the primary administrator is unavailable (34). Ensuring a secondary administrator is trained and available promotes consistency across the collection of PROMs and reduces the risk of missed data points when the primary administrator is unavailable (34). It is also important to anticipate ways the administrator may need to navigate supporting patient needs while also adhering to a study protocol. For example, providing common or standardized ways of explaining items would prepare an administrator to respond to patients who ask questions while completing a PROM. Providing guidance to all research personnel, particularly individuals interfacing with patients during data collection, not only improves the quality of data collected, but also promotes a positive environment for the research team, reducing stress and burden and promoting professionalism, and a participant population that is more informed about the process and potentially more likely to adhere to the research protocol (25, 34).

### Mode of PROM administration

Many PROMs were developed for paper-based administration, yet technological advances lead researchers to consider using electronic administration methods (e.g., RedCap, Qualtrics, OnlinePROMs). Research efforts comparing the paper-based and electronic modes of administration of patient responses (bias) has provided strong evidence demonstrating no difference in how patients respond when they complete PROMs *via* paper and electronic modes (2, 25, 39). While responses through paper-based or electronic methods are similar, there are considerations about response rate and cost of administration that may be factors in any assessment strategy (36, 40). Comprehensive strategies that combine electronic automated assessment, with human support such as checking for completeness of responses, reminders through postal mail or phone calls, and fielding email and phone inquiries from participants, have led to high response rates (36, 40). The cost-benefit of implementing a comprehensive strategy warrant more study. Regardless of how and where PROMs are administered, it is recommended that consistent administration methods of the same PROM be used within a study.

## PROM analysis and interpretation

One of the primary considerations with the use of PROMs in research and patient care is how to interpret and analyze scale scores to determine differences in groups and changes within patients over time. Interpretation is important to give context to the scores and to support clinical decision making. However, there are nuances around PROM score interpretation, such as response option scale and scoring, lack of data to inform individual score interpretation, confusion with change score concepts (12), and differences in methodology related to calculating change scores (11, 12, 41–45), that warrant consideration by researchers and may benefit from standardization when using these tools in sports medicine research.

When considering the formal statistical analysis of PROM data, researchers must consider the fundamental components of the PROM, just as they would with any other measurement tool. Specifically, researchers should consider the structure of response options for individual items and how they are rated by patients as well as how overall or total scores are calculated based on those ratings (17). For example, many region-specific PROMs [e.g., FAAM (46), FAST (47), LEFS (20)] ask the patient to rate each item on an ordinal scale, with those responses then converted into a total score that exists on a ratio scale (e.g., 0–100%). Awareness of these calculations is important because the scale will influence the type of statistical analysis appropriate for the data (e.g., parametric vs. non-parametric methods). Similarly, researchers should take care to ensure that PROM data meet basic assumptions required of a statistical test. For instance, previous investigations (48–50) have reported that PROMs data often demonstrate a skewed distribution and violate the basic assumption of normality. Skewed PROMs data are a particular concern in sport injury and rehabilitation research as the patient population is generally young and healthy, resulting in PROM scores skewed toward better scores or better reports of health status. The degree to which the data are skewed will influence the presentation of basic descriptive statistics, such as measure of central tendency (51). Medians and interquartile ranges are more appropriate for skewed data than are means and standard deviations (51). Further, skewed data may require non-parametric statistical tests or more complex modeling methods than data that are not skewed (52).

From a more clinically meaningful perspective, interpretation of scale scores is important for a single-point in time, such as return-to-play, and over-time, such as the time between the start of an intervention and end of an intervention, for the PROMs to have meaning in clinical practice or research (44, 53). The methods and values used to support these types of interpretation differ. With individual scores, reference or normative values from large patient populations may be helpful to gain a general understanding of whether a score is similar to those reported in a like-population (29, 44, 54), although some researchers suggest that comparisons of individuals to group level data be avoided (55). Knowing the representative values in the population of interest can give context to scores and supports interpretation. To date, there is limited information to inform score interpretation across the wide variety of instruments used in healthcare, including commonly used PROMs in sports medicine (16). Research is needed that aims to provide clinical meaning to individual scores and links scores to the characteristics of the population of interest. This type of research will help inform score interpretation and the meaning of research results. While interpretation of a score at one point in time is important, it is also essential to be able to interpret changes in scores over time.

Research that evaluates interventions is often analyzed using between group comparisons. While between group comparisons may apply to study aims, within-person or group change may better inform whether the intervention is having an impact over the longer-term. Within-person change is a fundamental characteristic with any health measurement, including PROMs, because knowledge of how scores change over-time within people and groups is important to correctly identify improvement, deterioration, or no change as a result of treatment or intervention. Values such as substantial clinical benefit (SCB) (56), minimal clinically important difference (MCID) (57) or minimal important change (MIC) (12, 53), and meaningful change threshold have been used to describe change in health status. Substantial clinical benefit is reflective of a larger or sustained patient-perceived improvement in health status whereas minimal changes reflect small but meaningful patient-perceived improvements in health status. Another concept that relates to change over time, but contributes to some confusion regarding score interpretation, is the minimal detectable change (MDC) which is a value that reflects the statistical error within a measurement and does not inform the clinical meaningfulness of a change score like SCB or MIC attempt to do (58). The MDC is valuable in determining whether change is within or outside of measurement error and is essential with all measurement tools. When interpreting research results over a period of time, researchers should determine whether their findings exceed the measurement error of the instrument (i.e., the MDC). Minimal detectable change (MDC) is based on statistical distributions and does not account for clinical factors. Therefore, efforts have been made to identify values, like SCB or MIC, that help researchers determine the clinical meaningfulness of research results.

Identifying a useful indictor of meaningful change relevant to PROMs is important because, in theory, measures of clinical meaningfulness should tie a clinical marker of health to patient perception of health. As previously mentioned, there are numerous values used to define clinically meaningfulness, and a preferred concept reflective of the smallest amount of change patients' perceive as beneficial is MIC (12, 44), although the more common term in the literature is MCID. Terwee et al. (12) emphasize how the words “change” and “minimal” are purposeful in the naming of MIC. Change helps to explain a within-person phenomena that speaks to a longitudinal assessment as opposed to the word “difference” which suggests a cross-sectional contrast between groups (12). “Minimal” highlights the value in small but meaningful changes in health status. While both longitudinal and between groups comparisons are valuable in interpreting PROMs, evaluation over time aligns with the primary purpose of healthcare which is to produce a change in patient health status, such as occurs from the transition from an injured to rehabilitated health state (59). Researchers should consider how data are reported related to the MIC. Use of proportions that reflect the number of patients in a group who exceeded MIC values is one recommendation that helps to illustrate the individual effect of an intervention within a group of patients and is easy to interpret (60, 61).

A challenge with using MIC to interpret PROMs is that given the methods of calculation, there is not a single MIC value for any PROM. Methods of calculation can include ROC analysis (12), adjusted ROC analysis (11, 41), predictive modeling (12, 45), as well as vignette-based methods (12). Factors such as severity of the health condition at baseline (11, 41), time points of intermittent PROM assessment (e.g., 1 week, 2 week, 1 month post-injury, or return-to-play), deteriorating or improving health state, choice of anchor (e.g., pain scale rating, global rating of change score, SANE rating), and definition of “minimal” are all likely to influence the resulting values calculated (13). Researchers who calculate MIC values should be detailed in their methods for increased transparency and with a goal of use in appropriate populations. However, the numerous ways to calculate meaningful change and the various factors that influence the calculation have called into question whether the MIC is as helpful in interpreting change in health status as it was initially thought to be (11, 41, 42). Future efforts by leaders in sports medicine research are needed to provide guidance on best practices related to calculating meaningful change scores. While the ROC method has been appealing given the inclusion of a clinical anchor and the ease of interpretation, efforts to improve on this methodology, such as with the adjusted ROC method, are promising and may be a step toward advancing the science of PROM interpretation (11, 41).

Finally, while efforts are ongoing to increase the interpretability of the change in PROMs scores over time, there are outcome measures about “health state” that are used as primary outcomes in sports medicine research (62–65). For example, the Patient Acceptable Symptom State (PASS) (63, 64, 66) gathers patient perspective about the satisfaction a patient has with their current health state, considering an acceptable level of symptoms, activities of daily living, and function that results in a satisfactory assessment of health. There are various version of the PASS, but most use a dichotomous response scale of “yes” or “no.” Describing the level of satisfaction a patient has with their state of health at the end of care or at the end of participation in a research study warrants consideration as a clinically meaningful research outcome (65, 67).

## Discussion

**Figure 1:**
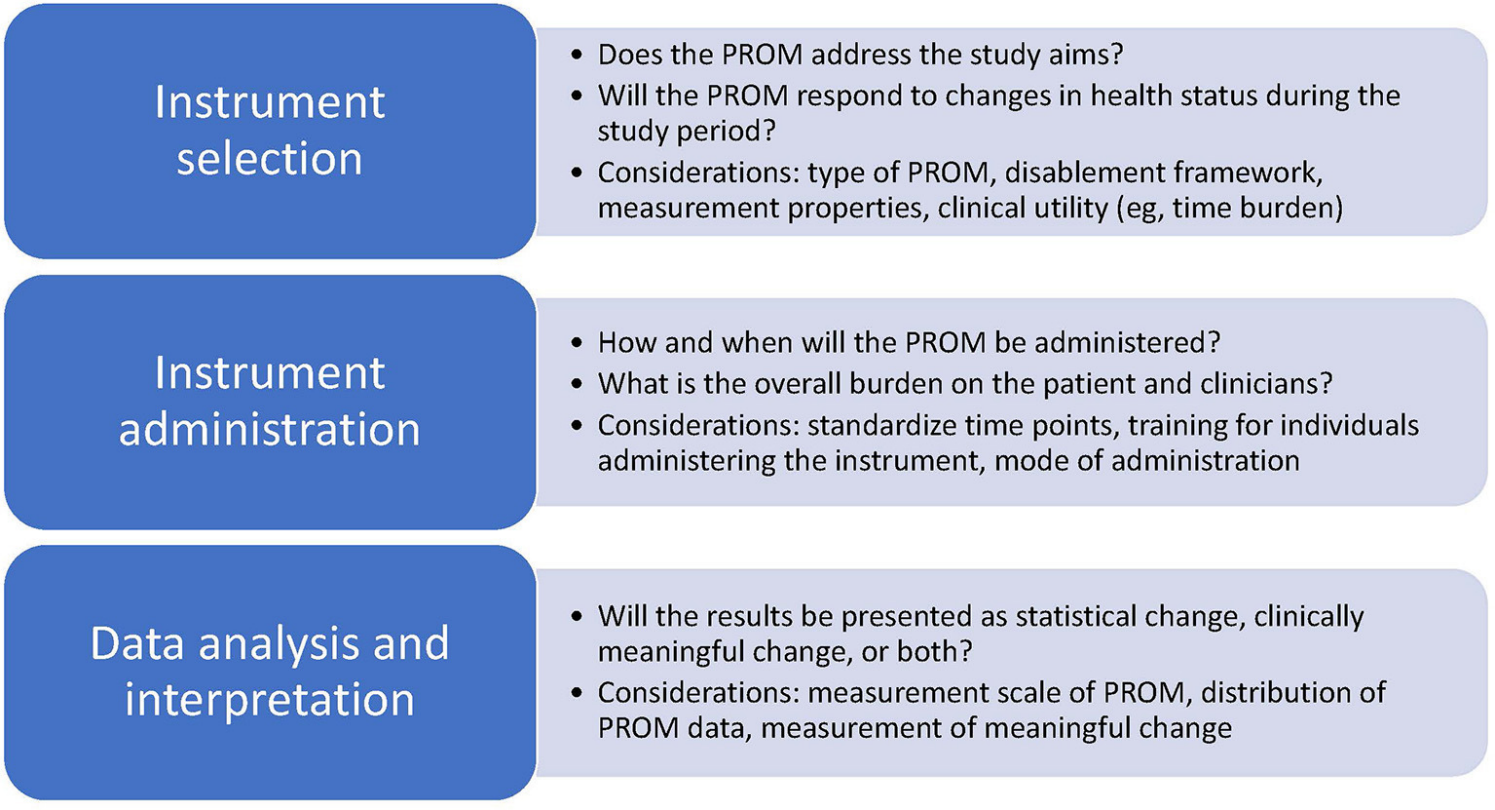
Considerations for instrument selection, administration, and analysis and interpretation for research using patient-reported outcome measures.

The future of sports medicine research is promising given the increased number of manuscripts with attention toward PROMs and the investigation in questions with patients at the center. The significant increase in attention toward person-centered variables such as quality of life, and efforts to evaluate health from a disablement model perspective, including evaluation of the impact of health conditions on social roles and environmental perspectives demonstrates the interest in conducting research with the patient at the center. Refinement of practices related to instrument selection, instrument administration, and data analysis and interpretation may support continued advancement in the quality, meaningfulness, and strength of the evidence used to promote the health of populations involved in sports medicine research (Figure 1). Additionally, increased awareness, collaboration, and adherence to national and international recommendations may support continued elevation of the quality of the science related to patient-oriented evidence (2, 4, 68). The National Institutes of Health (NIH), PCORI, and COSMIN group are three examples of entities that have prioritized patient-voice in research. Through their work, numerous lessons have been learned and advice and resources shared in an effort to improve collaborations and the quality of research using PROMs (Table 2) (2, 4, 68). Collectively, these leaders in healthcare have landed on similar recommendations, many of which highlight the critical importance of selecting PROMs and ensuring that they answer questions important to patients, that the assessment tools are administered within their limits, and that the outcomes have relevance and value to patients, clinicians, and researchers.

**Table 2:** Resources for using PROMs in sports medicine research.

**Resource**	**URL**
COSMIN Toolkit	https://www.cosmin.nl/
Patient-Centered Outcomes Research Institute	https://www.pcori.org/
NIH Common Data Elements EQUATOR Network Repository	https://cde.nlm.nih.gov/home equator-network.org
Registries	
AAOS Registry	https://www.aaos.org/registries/registry-program/about-the-aaos-registry-program/
NINDS CDEs	https://www.commondataelements.ninds.nih.gov/
Multicenter osteoarthritis study (MOST) public data sharing	https://most.ucsf.edu/multicenter-osteoarthritis-study-most-public-data-sharing
International Spinal Cord Injury (SCI) Core Data Sets	https://www.iscos.org.uk/international-sci-core-data-sets
Hip Fracture Network	https://fragilityfracturenetwork.org/what-we-do/hip-fracture-audit-database/
Federal Interagency Traumatic Brain Injury Research (FITBIR) Informatics System	https://fitbir.nih.gov/content/access-data
ICF-based documentation tool	https://www.icf-core-sets.org/

The notion of appropriateness of a PROM is a critical factor in advancing sports medicine research. Taking the time to select a PROM that has high relevance and meaningfulness to patients and clinicians may produce benefits to the success of the research. Using PROMs that are valid, reliable, and responsive to changes in patient care while also being of high relevance to patients and clinicians may strengthen adherence to the research protocol and the quality of the data collected. Over the last 20 years, there has been a significant increase in the body or research that include patient perspective and we look forward to the impact this research will have on the lives of patients.

## Author contributions

AS, KH, and TV contributed to conception and design of the study and wrote the first draft of the manuscript. AS, KH, KL, and TV wrote sections of the manuscript. All authors contributed to manuscript revision, read, and approved the submitted version.

## Conflict of interest

The authors declare that the research was conducted in the absence of any commercial or financial relationships that could be construed as a potential conflict of interest.

## Publisher's note

All claims expressed in this article are solely those of the authors and do not necessarily represent those of their affiliated organizations, or those of the publisher, the editors and the reviewers. Any product that may be evaluated in this article, or claim that may be made by its manufacturer, is not guaranteed or endorsed by the publisher.
